# Experimental determination of the energy difference between competing isomers of deposited, size-selected gold nanoclusters

**DOI:** 10.1038/s41467-018-03794-9

**Published:** 2018-04-03

**Authors:** D. M. Foster, R. Ferrando, R. E. Palmer

**Affiliations:** 1 Nanoscale Physics Research Laboratory, School of Physics and Astronomy, University of Birmingham, Birmingham, B15 2TT UK; 2 Chemistry and Industrial Chemistry Department, University of Genoa, Via Dodecaneso 31, 16146 Genoa, Italy; 3 College of Engineering, Swansea University, Bay Campus, Fabian Way, Swansea, SA1 8EN UK

## Abstract

The equilibrium structures and dynamics of a nanoscale system are regulated by a complex potential energy surface (PES). This is a key target of theoretical calculations but experimentally elusive. We report the measurement of a key PES parameter for a model nanosystem: size-selected Au nanoclusters, soft-landed on amorphous silicon nitride supports. We obtain the energy difference between the most abundant structural isomers of magic number Au_561_ clusters, the decahedron and face-centred-cubic (fcc) structures, from the equilibrium proportions of the isomers. These are measured by atomic-resolution scanning transmission electron microscopy, with an ultra-stable heating stage, as a function of temperature (125–500 °C). At lower temperatures (20–125 °C) the behaviour is kinetic, exhibiting down conversion of metastable decahedra into fcc structures; the higher state is repopulated at higher temperatures in equilibrium. We find the decahedron is 0.040 ± 0.020 eV higher in energy than the fcc isomer, providing a benchmark for the theoretical treatment of nanoparticles.

## Abstract (web summary)

The equilibrium structures and dynamics of a nanoscale system are regulated by a complex potential energy surface (PES), a key target of theoretical calculations but experimentally elusive. Here, the authors report the measurement of a key PES parameter for size-selected Au nanoclusters soft-landed on amorphous silicon nitride supports.

## Introduction

The structure and dynamics of a nanosystem are controlled by the multi-dimensional potential energy surface (PES), which describes its free energy as a function of configuration. There have been considerable theoretical efforts to determine the ground-state structures and energy differences between competing isomers of nanosytems in general^1–3^ and of nanoclusters in particular^4–8^. Gold clusters have received much theoretical attention due to the role of structure in the catalytic performance^9^. What is needed now is an experimental handle on key parameters of the PES. Understanding the energy difference between structural isomers is important not only for the design of well-defined materials but also for understanding how these materials will work in situ. For example, if a particular structural isomer is unstable, exposure to high temperatures is likely to drive it towards the ground state (i.e. annealing), altering (for better or worse) the characteristics of the system. Such behaviour is likely to be relevant to the applications of nanoparticles, which include catalysis^10,11^, drug delivery^12,13^ and chemical sensing^14^.

Experimentally the atomic structure of nanoclusters can be determined, to various degrees, by a number of techniques including trapped ion electron diffraction^15^, x-ray scattering^16^, transmission electron microscopy (TEM) tilt series^17^ and high-angle annular dark-field (HAADF) aberration-corrected scanning transmission electron microscopy (ac-STEM)^18^. However, the cluster formation conditions can easily lead to the trapping of higher lying isomers and the populations of cluster isomers observed do not represent thermal equilibrium^19^. Previous STEM studies have provided some qualitative insight into the PES of clusters through e-beam transformation experiments. By continual imaging during intense irradiation, Au_561_^20^ and Au_923_^19^ clusters (on carbon) have been shown to transform one-way to lower energy structures^19^, while smaller clusters fluctuate continually^21–23^. Such experiments enable candidate low energy structures to be identified. However, these experiments do not provide the quantitative energy difference between isomers. Ex situ annealing experiments by Koga et al.^24^ found that annealing of small and medium sized (<14 nm) Au clusters below the melting point (<1273 K) resulted in structural transformations, but no quantitative measure of energy differences or barrier heights could be made.

Here we employ a precision heating stage in ac-STEM to determine in situ the proportion of structural isomers for size-selected Au_561_ clusters, deposited on amorphous silicon nitride, over a range of temperatures. This enables the energy difference between competing fcc and Dh isomers in the equilibrium region to be extracted for Au_561_ on the surface. We identify two regimes: a low-temperature regime in which metastable (kinetically trapped) Dh clusters transform to fcc, and a high-temperature regime in which the Dh isomer is repopulated (Boltzmann statistics); here the system is in thermal equilibrium. From the equilibrium, high-temperature region data, we find that the Dh and fcc isomers are very close in energy, where the Dh are only 0.040 ± 0.020 eV higher than those of fcc.

## Results

### Electron microscope images

**Fig. 1:**
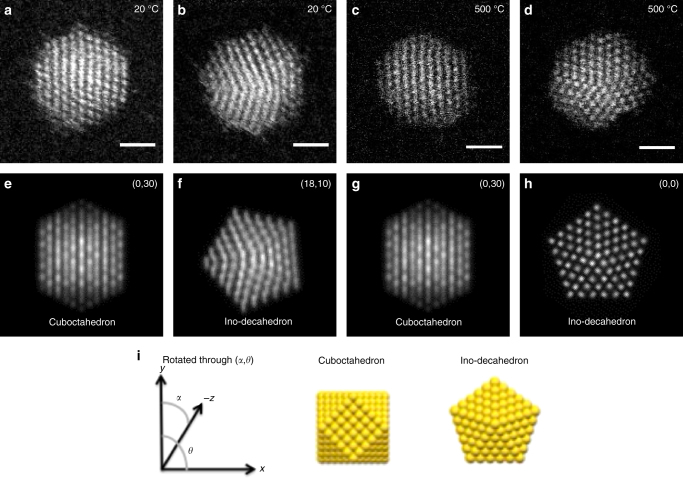
HAADF STEM images of Au_561_ clusters at 20 °C and 500 °C. **a**–**d** HAADF STEM images of Au_561_ clusters and **e**–**h** matching multi-slice electron scattering simulations of the cuboctahedron and Ino-decahedron at different orientations. **a**, **b** Experimental images recorded at 20 °C; **c**, **d** Images recorded at 500 °C. **i** Rotation angle of the cuboctahedron and Ino-decahedron geometries

Figure 1 shows examples of HAADF STEM images of Au_561_ clusters on amorphous silicon nitride and corresponding multi-slice simulations from a simulation atlas^19^. Figure 1a and b was recorded at 20 °C. Figure 1a shows an fcc cluster and Fig. 1b shows a decahedral cluster. Figure 1c and d was recorded at 500 °C. Figure 1c shows an fcc cluster and Fig. 1d an on-axis decahedron. Both decahedra in Fig. 1b and d show some Marks reentrant features. In comparison of experimental and simulated images, we concentrate on the core atomic structure because this is where the signal-to-noise levels are the highest, so that we can compare them with simulations of perfect cuboctahedra and Ino-decahedra. HAADF STEM images matched to the cuboctahedron simulations are denoted face-centred-cubic (fcc), which allows for variation in the exact surface truncation; similarly images matched to the Ino-decahedron are denoted decahedra (Dh). Clusters that display ‘ring-dot’ features in the images, a characteristic of an icosahedron, are denoted simply as icosahedra (Ih).

### Proportions of different isomers

**Fig. 2:**
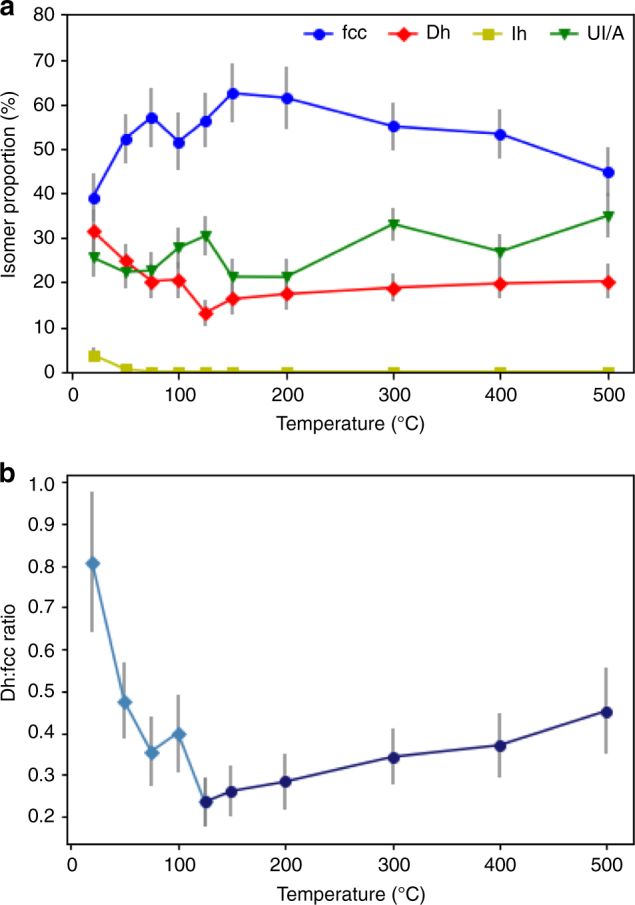
The proportion of structural isomers versus temperature. **a** The proportion of structural isomers for Au_561_ clusters on amorphous silicon nitride at ten temperatures: 20 °C, 50 °C, 75 °C, 100 °C, 125 °C, 150 °C, 200 °C, 300 °C, 400 °C and 500 °C. The clusters are classified as face-centred-cubic (circles), decahedral (diamonds), icosahedral (squares) or unidentified/amorphous (triangles). The numbers of experimental images recorded at each temperature are 133, 161, 128, 126, 151, 141, 132, 191, 167 and 143 respectively. Poisson error bars, derived from these statistics, are shown for the isomer proportions. **b** The ratio of Dh to fcc clusters versus temperature. The low-temperature regime (20–125 °C) is in diamond markers and the high-temperature regime (125–500 °C) in circle markers. Lines between points plotted are simply a guide to the eye. Error bars, derived according to the error propagation law, are shown for the Dh:fcc ratio

Figure 2a is a plot of the proportions of structural isomers, extracted from the fits to the experimental data, for Au_561_ clusters on amorphous silicon nitride at temperatures ranging from 20 °C to 500 °C. The same sample was used for all measurements so that formation conditions would not affect the results^25^. Cluster structures are identified as either fcc, Dh, Ih or unidentified/amorphous (UI/A). The error bars on the proportions of structural isomers are statistical counting errors and the error on the temperature is 5%, due to the heating chip calibration. At all temperatures investigated the most abundant isomer is fcc, followed by Dh, while Ih has a very low abundance (0–3%). We find that the clusters still provide a good match with the simulated structures at high temperature and there is no evidence of melting in the temperature range explored here, as can be seen from Fig. 1. The percentage of unidentified or unknown (UI/A) structures—clusters that are amorphous or cannot be identified using simulation atlases for the Ino-decahedron, cuboctahedron or icosahedron—is fairly constant across the temperature range. One explanation for such images is that only single-shot data was taken (to minimise the electron dose), and clusters often rotate during scanning.

Figure 2b shows a plot of the ratio of the two most abundant ordered isomers, Dh and fcc, versus temperature. Two distinct temperature regimes are clearly visible. Between 20 °C and 125 °C the Dh:fcc ratio decreases from 0.81 to 0.24, whereas between 125 °C and 500 °C the Dh:fcc ratio increases from 0.24 to 0.45. The underlying and associated errors are derived from Fig. 2a. Between 20 °C and 150 °C the increase in temperature results in an increase in the abundance of the fcc isomer, but at temperatures ≥150 °C the proportion of fcc gradually decreases again. Complementary to this, between 20 °C and 125 °C, the proportion of Dh decreases, whereas at temperature ≥125 °C there is a slight increase in Dh as temperature rises.

The increase in the proportion of fcc clusters from 20 °C to 125 °C, and the corresponding decrease in the proportion of Dh, can be explained in terms of the release of trapped metastable Dh structures to a lower free energy fcc structure. We previously reported that Au_561_ clusters undergo a one-way transition from Dh to fcc when continuously exposed to the STEM electron beam at very high magnification^20^, which corresponds to moderate heating of the sample. However, the behaviour we observe takes on a new character above 125 °C with the ratio of Dh to fcc increasing again.

This repopulation behaviour can be understood if the fcc structure is a lower free-energy structure than the Dh. Then, beyond the release of kinetically trapped Dh clusters by annealing at temperatures from 20 °C to 125 °C, we may expect that an equilibrium distribution of isomers will be established at higher temperatures. A proportion of the clusters (based on Boltzmann statistics) will be excited from the fcc to the higher energy Dh structure^26^. In fact, if we assume equilibrium between isomers of energy *E*_Dh_ and *E*_fcc_, we obtain the ratio between the probabilities *p*_Dh_ and *p*_fcc_ of the corresponding structures given by (see the Supplementary Note 1 for a derivation of this formula)

1 $${\mathrm{ln}}\left( {p_{{\mathrm{Dh}}}/p_{{\mathrm{fcc}}}} \right) = \beta (E_{{\mathrm{fcc}}} - E_{{\mathrm{Dh}}}) + c$$

where *β* = (*k*_B_*T*)^−1^.

In this system the Ih must have much higher energy, as we do not see repopulation of this isomer even at 500 °C; this is in agreement with experimental observations of Ih Au_923_ clusters under the electron beam, which transformed to Dh or fcc structures after very short exposure times^19^. If the increase in the proportion of Dh clusters in the high-temperature region is a result of thermal repopulation of this excited state, the energy difference between the Dh and fcc structural isomers can be derived, as we show below. A second hypothetical explanation for the change in ratio is that, as the temperature of the clusters increase, atoms are lost through sublimation resulting in a smaller cluster size at higher temperatures where the decahedron might in principle be more stable. However, based on analysis of the diameters of the clusters at 500 °C (Fig. 1), we are confident that no major loss of atoms has occurred.

**Fig. 3:**
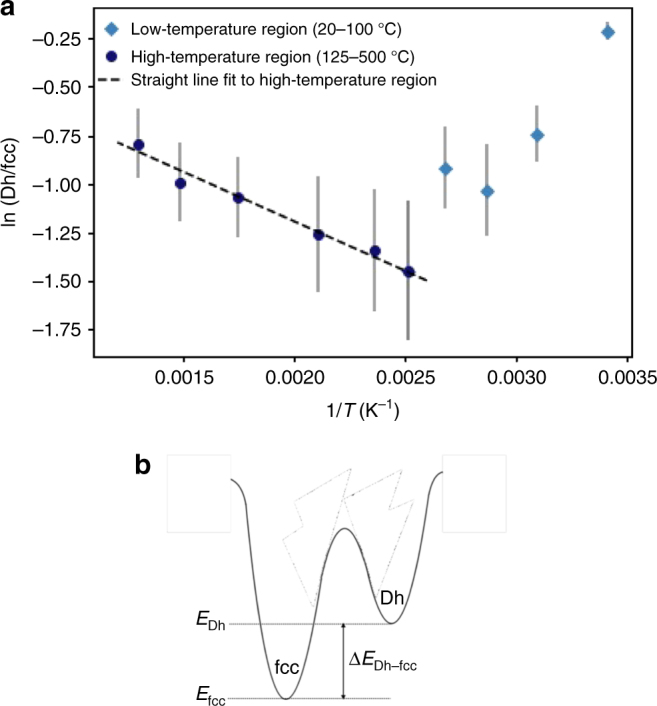
The derived energy difference between fcc and Dh minima. **a** Ratio of the Dh to fcc abundances (natural log plot) for Au_561_ plotted against the reciprocal of the temperature (in Kelvin). The dashed line shows a weighted least squares fit to the high-temperature region. The equation of this line is: *y* = *mx* + *c* where *m* = −510240 K and *c* = −0.20 ± 0.40 in units of *k*_B_. Error bars, derived from data in Fig. 2 and using error propagation laws, are shown for the natural log of the Dh:fcc ratio. **b** A schematic of the derived energy difference between fcc and Dh minima

Figure 3 shows a plot of the natural log of the ratio of the Dh and fcc abundances as a function of the reciprocal of the absolute temperature. From Eq. (1), the slope of the line in the higher temperature equilibrium regime gives the energy difference between the local minima of the two competing isomers, whereas the intercept gives the entropy difference (see Supplementary Note 1 for detailed explanation). This does not apply to the low temperature, kinetic regime. The dashed line shows a weighted linear least squares fit to the high-temperature region (398–773 K) of the plot. The gradient of this line is −510 ± 240 K, which corresponds to a value of 0.040 ± 0.020 eV (*E* = *k*_B_*T*) for the energy difference between Dh and the lower lying fcc isomers (Δ*E*_Dh–fcc_). The intercept *c* = −0.2 ± 0.4 is the entropy difference in units of *k*_B_ (Supplementary Note 1), which indicates a negligible entropy difference between these structures.

## Discussion

There are two key assumptions that underpin these new results. First, the partition function for each isomer is given by the harmonic superposition approximation, in which the vibrational frequencies are assumed to be harmonic and independent of temperature. In many cases this approximation has been shown to be valid for temperatures below the melting point^8^. If the vibrational frequencies are anharmonic, there would be a temperature dependence^8^, possibly resulting in non-linearity in the plot of ln(Dh/fcc) versus 1/*T*. Secondly, we have assumed that for each basin (Dh-basin, fcc-basin) in the PES of the cluster, there is only a contribution from one structural isomer. In the experiment we have a small range of cluster sizes (determined by the mass filter resolution), and within the classification of Dh or fcc there may be different truncations and arrangements of atoms on the surface that are not easily distinguished by our simulation atlas method. If whole ‘families’ of Dh and fcc isomers are being observed experimentally, the energy difference determined may represent a sort of weighted average of the energy differences between the Dh and fcc clusters in the families. However, in this case there would not be any compelling reason for obtaining the linear increase shown in Fig. 3 for high temperatures.

The derived energy difference of 0.04 eV between the Dh and fcc isomers is very small (corresponding to only ≈510 K), and means that at the cluster size investigated, 561 ± 14, these isomers compete very closely. Of course it is the closeness in energy that makes the energy difference nicely measureable in our experiment, which probes a temperature range of 375 °C (125–500 °C). Regarding the derived energy difference, it would be appealing at this point to produce a theoretical calculation that predicts an energy offset comparable with the experimental value. But the truth is that no calculations currently offer accuracy at the level of tens of meV for hundreds of atoms! However, the result is in broad qualitative agreement with several^27–29^ theoretical calculations that predict Dh and fcc isomers competing in energy at this size range. The original molecular dynamic simulations for Au clusters by Baletto et al.^27^ used the second-moment tight-binding potential for a detailed study and EAM potentials to determine general trends in the energetics of icosahedra, decahedra and truncated octahedral clusters. They found a crossover size from Dh to fcc at 500 atoms, above which the Dh and fcc isomers remain close in energy, whilst the Ih is not favoured above 100 atoms. More recently, Wang et al.^28^ also found that fcc is the lowest energy structure for clusters with more than 500 atoms; between 500 and 2000 atoms the truncated octahedron was their lowest energy structure followed by octahedron, truncated decahedron and Ih. In this case calculations were performed using Ino’s theory with parameters from the Sutton-Chen potential. DFT calculations performed by Li et al.^29^ showed that for Au_561_, the order of stability was fcc, Dh and Ih. In contrast to these results, Barnard et al.^30^ reported that, based on a thermodynamic model, the Ih was the most stable structure at room temperature, while at temperatures comparable to our equilibrium region the Dh was the most stable structure. In both cases fcc was the lowest free energy structure only for cluster sizes >15 nm. In a global optimisation study (using the RGL potential) by Göedecker et al.^31^, it was found that a truncated octahedral Au cluster with 201 atoms was only 0.007 eV higher in energy than a 192 atom Marks decahedral cluster. These very small differences in energy between Dh and fcc isomers are broadly consistent with our experimental observations.

Given the small energy difference obtained between the two principal isomers, 40 meV, the influence of the substrate needs to be considered. As described in Note 2 of the Supplementary Information, we have conducted an experimental investigation of the same isomers of Au_561_ but this time on an amorphous carbon support. The behaviour observed is similar: an annealing regime followed by an equilibrium regime; the fcc structure has the lowest energy; the Dh is, in this case, found to lie 20 meV higher in energy. We conclude that the method reported has general applicability to different systems and that the change of support does not markedly alter the relative energies. Another question is: does the surface switch the relative stability of the two isomers compared with the free clusters? Without any experimental data from the gas phase one cannot be sure, but we have conducted a theoretical treatment of the substrate effect (Supplementary Note 3). This shows that a model of the carbon surface has a tendency to stabilise the fcc isomer more than it does the Dh isomer, which relates to the facet sizes in contact with the support. Thus it is possible that the favoured configuration of a free cluster could switch on the surface from Dh to fcc.

In summary, we have demonstrated a method to obtain experimentally a critical parameter in the PES of a model nanosystem. Specifically, we have reported the proportions of competing structural isomers as a function of temperature in a population of model size-selected Au_561_ clusters, soft-landed on amorphous silicon nitride. The approach employs atomic-resolution imaging with an ultrastable heating stage in the aberration-corrected STEM. Two distinct kinds of behaviour have been identified. In the low-temperature region, from 20 to 125 °C, there is a decrease in the Dh:fcc ratio, attributed to the transformation of kinetically trapped metastable Dh into lower energy fcc structures. In the higher temperature region, from 125 to 500 °C, the Dh:fcc ratio increases; the Dh isomer is repopulated because the system is in equilibrium. The measured equilibrium populations enable us to determine the energy difference between the two isomers. We find that the Dh isomer is 0.040 ± 0.020 eV higher in energy than the fcc for Au_561±14_. Ultimately, such quantitative parameters of the PES allow for a direct comparison with, and benchmark of, theoretical treatments and thus a new insight into the equilibrium structures and dynamics of nano-systems.

## Methods

### Cluster deposition

Au clusters consisting of 561 ± 14 atoms were produced with a magnetron sputtering gas aggregation cluster beam source^32^, incorporating a lateral time of flight mass filter (*M*/Δ*M* = 20)^33^. The clusters were deposited onto the amorphous silicon nitride films of the heating chips in the soft-landing regime (<2 eV/atom)^34^ to preserve their original atomic structure.

### Electron microscopy

A JEOL 2100F STEM with spherical aberration probe corrector (CEOS) was employed for atomic-resolution imaging of the nanoclusters. The convergence angle was 19 mrad, and the inner and outer HAADF detector collection angles were 62 mrad and 164 mrad respectively. In situ heating was performed using a heating holder with MEMS-based heating chips (DENS Solutions). The chips consist of a metal heater coil embedded in silicon nitride, surrounded by imaging windows of amorphous silicon nitride. A current is applied to the metal coil to heat the chips: the temperature comes from the resistance measured in situ using the four-point probe method, with chip calibration performed by the supplier. The error on the temperature measurement is 5% (a potential systematic error) and the temperature stability <1 °C. Experiments were conducted by setting the temperature to a chosen value and taking single-shot HAADF STEM atomic-resolution images of a population of clusters. The temperature was increased incrementally (from 20 °C to 500 °C) and at each temperature ≥100 clusters were imaged. The atomic structures of the individual clusters were then identified by comparison with multi-slice electron scattering simulations of the (unsupported) cuboctahedron, Ino-decahedron and icosahedron isomers at different orientations (polar and azimuthal) using the QSTEM package and the simulation atlas method^19^.

### Data availability

All data is available from the authors upon reasonable request.

## Electronic supplementary material

Supplementary Information (PDF 767 kb)
