# Supplementary material for: Experimental determination of the energy difference between competing isomers of deposited, size-selected gold nanoclusters
Source: Nat Commun. 2018 Apr 3;9:1323. doi: 10.1038/s41467-018-03794-9 (PMC5882772; doi:10.1038/s41467-018-03794-9)
Supplement: Supplementary file 1 — Supplementary Information (PDF 767 kb) [file 41467_2018_3794_MOESM1_ESM.pdf]

Experimental determination of the energy difference between competing isomers of deposited, size-selected gold nanoclusters

Foster et al.

Note 1 – Probabilities of isomers in the harmonic superposition approximation

For a cluster of size N, in the superposition approximation, the partition function Z can be written as

$$Z = \sum_i Z_i \tag{1}$$

where Z_i is the partition function of the i-th local minimum on the potential energy surface[1,2]. The probability p_i of isomer i is given by

$$p_i = \frac{Z_i}{Z}. \tag{2}$$

Z_i can be factored into translational, rotational, and vibrational partition functions

$$Z_i = Z_i^{\mathrm{tr}} Z_i^{\mathrm{rot}} Z_i^{\mathrm{vib}}\, \mathrm{e}^{-\beta E_i}, \tag{3}$$

where $\beta = 1/(k_\mathrm{B}T)$ and E_i is the energy of local minimum i.

Let us consider an fcc isomer and a Dh isomer. The ratio of their probabilities is therefore given by

$$\frac{p_\mathrm{Dh}}{p_\mathrm{fcc}} = \frac{Z_\mathrm{Dh}}{Z_\mathrm{fcc}}. \tag{4}$$

Since we are interested in temperature above the Debye temperature of Au, we assume the classical approximation to Z. We note that Z_{tr} is the same for all isomers, and neglect differences between the rotational partition functions, since they give minor contributions[2]. According to these approximations it turns out that

$$\frac{p_\mathrm{Dh}}{p_\mathrm{fcc}} = \frac{n_\mathrm{Dh}\, \mathrm{e}^{-\beta E_\mathrm{Dh}}\, \overline{\omega}_\mathrm{fcc}^{3N-6}}{n_\mathrm{fcc}\, \mathrm{e}^{-\beta E_\mathrm{fcc}}\, \overline{\omega}_\mathrm{Dh}^{3N-6}}. \tag{5}$$

The multiplicities n_Dh and n_fcc are given by

$$n_\mathrm{Dh} = \frac{2N!}{g_\mathrm{Dh}} \qquad n_\mathrm{fcc} = \frac{2N!}{g_\mathrm{fcc}} \tag{6}$$

where g_Dh and g_fcc are the orders of the symmetry groups of the two isomers. Therefore

$$\frac{p_\mathrm{Dh}}{p_\mathrm{fcc}} = \frac{g_\mathrm{fcc}\, \mathrm{e}^{-\beta E_\mathrm{Dh}}\, \overline{\omega}_\mathrm{fcc}^{3N-6}}{g_\mathrm{Dh}\, \mathrm{e}^{-\beta E_\mathrm{fcc}}\, \overline{\omega}_\mathrm{Dh}^{3N-6}}, \tag{7}$$

which gives

$$\ln\left(\frac{p_\mathrm{Dh}}{p_\mathrm{fcc}}\right) = \frac{1}{k_\mathrm{B}T}(E_\mathrm{fcc} - E_\mathrm{Dh}) + \ln\left(\frac{g_\mathrm{fcc}\, \overline{\omega}_\mathrm{fcc}^{3N-6}}{g_\mathrm{Dh}\, \overline{\omega}_\mathrm{Dh}^{3N-6}}\right) \tag{8}$$

If both isomers are of low symmetry ($g_\mathrm{Dh} = g_\mathrm{fcc} = 1$) then

$$\ln\left(\frac{p_\mathrm{Dh}}{p_\mathrm{fcc}}\right) = \frac{1}{k_\mathrm{B}T}(E_\mathrm{fcc} - E_\mathrm{Dh}) + (3N-6)\, \ln\left(\frac{\overline{\omega}_\mathrm{fcc}}{\overline{\omega}_\mathrm{Dh}}\right). \tag{9}$$

The intercept c in the Arrhenius plot is thus related to the logarithm of the

ratio of the average vibrational frequencies.

We can relate it to the entropy difference between the isomers. In fact

$$\Delta F = F_{\rm Dh} - F_{\rm fcc} = \Delta E - T\Delta S. \tag{10}$$

But

$$\Delta F = -k_{\rm B}T \ln\left(\frac{Z_{\rm Dh}}{Z_{\rm fcc}}\right) = -k_{\rm B}T \ln\left(\frac{p_{\rm Dh}}{p_{\rm fcc}}\right). \tag{11}$$

From Supplementary Equations (9,10,11) follows that

$$\Delta S = ck_{\rm B}. \tag{12}$$

Therefore, within the assumption of low symmetry, the intercept is the vibrational entropy difference between these isomers in units of $k_{\rm B}$. If low symmetry does not hold, the intercept still corresponds to the entropy difference, but in this case the difference includes the contribution from configurational factors.

Note 2 – Experimental data for Au_{561} on amorphous carbon

In these additional experiments, the method was exactly the same as in the main paper. The only differences are the support, the range of temperatures investigated, and the quantity of data recorded. The cluster structures observed are illustrated in Supplementary Figure 1

Supplementary Figure 2 shows the proportions of structural isomers observed for Au_{561} clusters on a-carbon at temperatures ranging from 20 to 250 ^{o}C. The data at 20 ^{o}C are taken from Ref. [3] in which exactly the same cluster formation conditions were used. Cluster structures are identified as either face-centred-cubic (fcc), decahedral (Dh), icosahedral (Ih) or unidentified/amorphous (UI/A). The errors in the proportion of structural isomers are statistical counting errors, and the error on the temperature is 5% - this is due to the heating chip calibration. At both 20 and 50 ^{o}C the majority of structures are Dh, at temperatures greater than 50 ^{o}C there is a crossover and the majority of structures are fcc. In all cases the proportion of Ih is very low (0-3%). The percentage of UI/A structures is fairly constant across the temperature range. We find that the clusters still provide a good match with the simulated structures at high temperature, and there is no evidence of melting, as can be seen from Supplementary Figure 1.

The data shown in Supplementary Figure 2 (see also Supplementary Figure 3) can be interpreted in the same way as the data for Au_{561} on silicon nitride in the main paper. In the low temperature region (20-100 ^{o}C), the increase in fcc and corresponding decrease in Dh can be interpreted as the release of trapped metastable Dh. In the high temperature region (100-250 ^{o}C) we assume the system is in equilibrium, and the increase in Dh (and decrease in fcc) is attributed to the excitation of low energy fcc structures into higher energy Dh structures.

Supplementary Figure 3 shows a plot of the natural log of the ratio of Dh

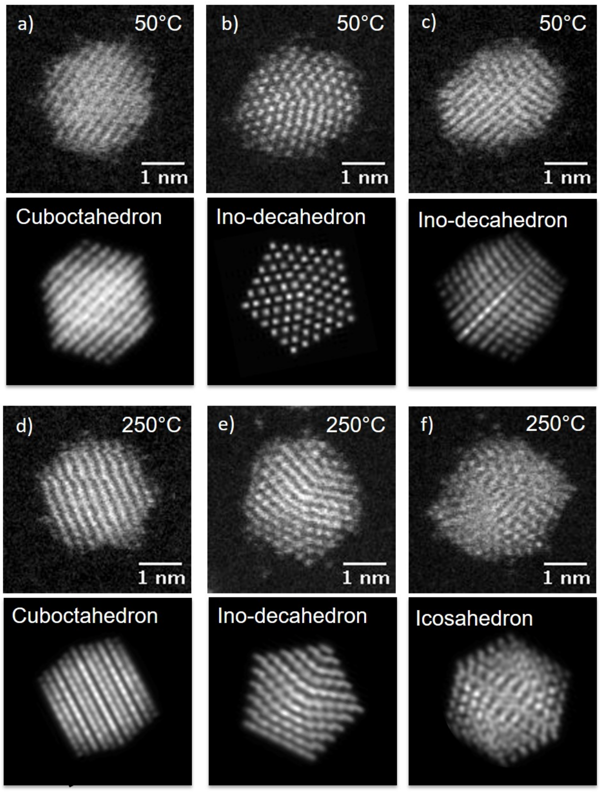

Supplementary Figure 1: HAADF STEM images of Au_{561} clusters and matching multi-slice HAADF STEM simulations of the cuboctahedron, Ino-decahedron, and icosahedron at different orientations. a)-c) show images recorded at 50 ^{o}C and d)-f) show images recorded at 250 ^{o}C.

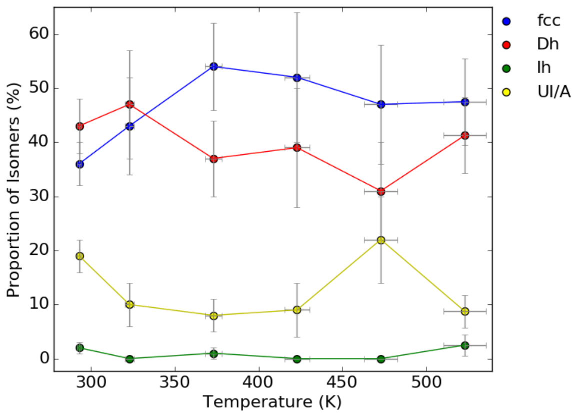

Supplementary Figure 2: The proportion of structural isomers for Au_{561} at 20 , 50 , 100 , 150 , 200 and 250 ^{o}C. The clusters are classified as either face-centred-cubic (blue), decahedral (red), icosahedral (green) or unidentified/amorphous (yellow). The numbers of experimental images recorded at each temperature are 189, 51, 76, 33, 36 and 80 respectively. Poisson error bars are shown. The bars on the temperature axis correspond to a 5% error.

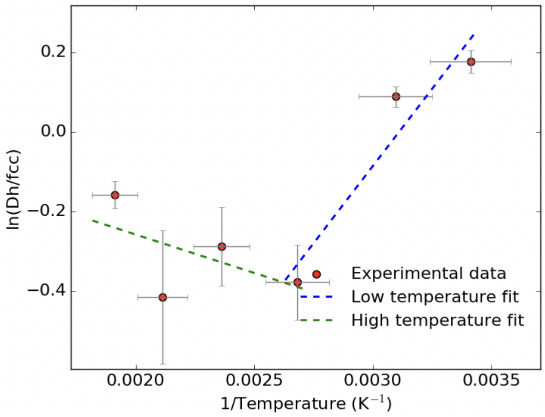

Supplementary Figure 3: Ratio of the Dh to fcc abundances (natural log plot) for Au_{561} plotted against the reciprocal of the temperature (in Kelvin). Scatter points show measurements taken at 20, 50, 100, 150, 200 and 250 ^{o}C. A fit to the low temperature region, 20-100 ^{o}C, is shown by a blue line. A fit to the high temperature region, 100-250 ^{o}C, is shown by a green line. The numbers of experimental images recorded at each temperature are 189, 51, 76, 33, 36 and 80 respectively. Poisson error bars are shown. The bars on the temperature axis correspond to a 5% error.

and fcc abundances as a function of the reciprocal of the absolute temperature. Based on the gradient of the line fit to the high temperature data, a value of 0.02 eV is obtained for the energy difference between Dh and the lower lying fcc isomers, a value that lies within experimental error of the result on silicon nitride of the main paper.

Comparison between the data for a-carbon and silicon nitride supports shows that the observed behavior is almost identical, and confirms the generality of the method reported in the paper. Though the data on the carbon support is more limited at this stage (the number of clusters imaged is lower and the range of temperature investigated smaller), it seems as if the preference of Au_{561} clusters for the fcc structure in equilibrium is not sensitive to the precise details of the substrate. There is a small difference in the initial proportions of Dh and fcc clusters (a-carbon, fcc 36% and Dh 43%; silicon nitride: fcc 39% and Dh 31%). This may be a result of marginally different formation conditions (e,g, temperature in the condensation chamber) or of the change in support.

Note 3 – Supported clusters on a flat surface

We consider a homogeneous half-space filled of Lennard-Jones centers to mimic the qualitative effect of a substrate interacting with Au by means of van der Waals forces. This is a completely smooth and flat substrate, which is likely to overestimate the interaction between the substrate and the deposited clusters as regards the adhesion of extended facets (localized defects are likely to bind in the same way to such large clusters, whatever the structure).

Let us start from a Lennard-Jones potential for the interaction between an Au and a C atom at distance x

$$U(x) = 4\varepsilon \left(\frac{\sigma^{12}}{x^{12}} - \frac{\sigma^6}{x^6} \right) \tag{13}$$

If we assume that the C substrate is made of a continuous distribution of Lennard-Jones centers, after integration one finds[4] an effective substrate potential U_{sub}, which depends only on the distance z between the Au atom and the substrate surface:

$$U_{\text{sub}}(z) = \frac{C_9}{z^9} - \frac{C_3}{z^3} \tag{14}$$

with

$$C_3 = \frac{2\pi}{3}\rho_{\text{sub}}\varepsilon\sigma^6 \qquad C_9 = \frac{4C_3^3}{27D^2} = \frac{4\pi}{45}\rho_{\text{sub}}\varepsilon\sigma^{12} \tag{15}$$

The force is given by

$$F(z) = -\frac{dU}{dz} = 9\frac{C_9}{z^{10}} - 3\frac{C_3}{z^4} \tag{16}$$

which gives zero when

$$z^* = \left(\frac{3C_9}{C_3} \right)^{1/6} \tag{17}$$

which corresponds to the well depth D

$$D = -U(z^*) = \frac{2}{3^{3/2}} \frac{C_3^{3/2}}{C_9^{1/2}} \tag{18}$$

We assume that the total interaction between the cluster and the substrate is given by a sum of single-atom contributions.

Now we consider the quantitative aspects. The density of graphite $\rho_{\rm sub}$ follows from the fact that the unit cell is a rhombic prism of height $c = 6.694\,\text{Å}$ and of rhombus side $a = 2.456\,\text{Å}$. The unit cell contains 4 carbon atoms. Therefore

$$\rho_{\rm sub} = 0.11439\,\text{Å}^{-3}. \tag{19}$$

Now we consider two different choices of the parameters ε and σ of the Au-C Lennard-Jones potential from the literature.

P1. From Ref. [5]

$$\varepsilon = 0.01273\,\text{eV} \qquad \sigma = 2.9943\,\text{Å} \tag{20}$$

which gives

$$C_3 = 2.198\,\text{eV}/\text{Å}^3 \qquad C_9 = 211.231\,\text{eV}/\text{Å}^9. \tag{21}$$

With this choice one has, for a single adsorbed atom,

$$z^* = 2.57\,\text{Å} \qquad D = 0.0863\,\text{eV}. \tag{22}$$

P2. From Ref. [6]

$$\varepsilon = 0.022\,\text{eV} \qquad \sigma = 2.74\,\text{Å} \tag{23}$$

which gives

$$C_3 = 2.230\,\text{eV}/\text{Å}^3 \qquad C_9 = 125.839\,\text{eV}/\text{Å}^9. \tag{24}$$

With this choice one has, for a single adsorbed atom,

$$z^* = 2.35\,\text{Å} \qquad D = 0.114\,\text{eV}. \tag{25}$$

The Au-Au interaction is modelled as in Ref. [3].

Now we compare the best truncated octahedron (TO) and decahedron (Dh) in free space (as obtained by global optimization searches) and supported on the substrate for Au_{561} (see Supplementary Figure 4. Supported clusters are obtained by keeping the same structure as in free space and depositing them in such a way that they adhere to the substrate by their larger facet (which is a (111) facet in both cases). The energy differences between fcc and Dh structures are reported in Supplementary Table 1. In all cases, fcc are higher in energy, so that we report $\Delta E = E_{\rm fcc} - E_{\rm Dh}$. which is positive

The results for free clusters do not agree with the experimental data, in the sense that they predict the Dh to be lower in energy. Since energy differences are quite small (1 meV/atom or less), it is very difficult to obtain an agreement with the experiments. However one result emerging is that, while the effect of the substrate is modest, it produces a larger stabilising effect for the fcc structure

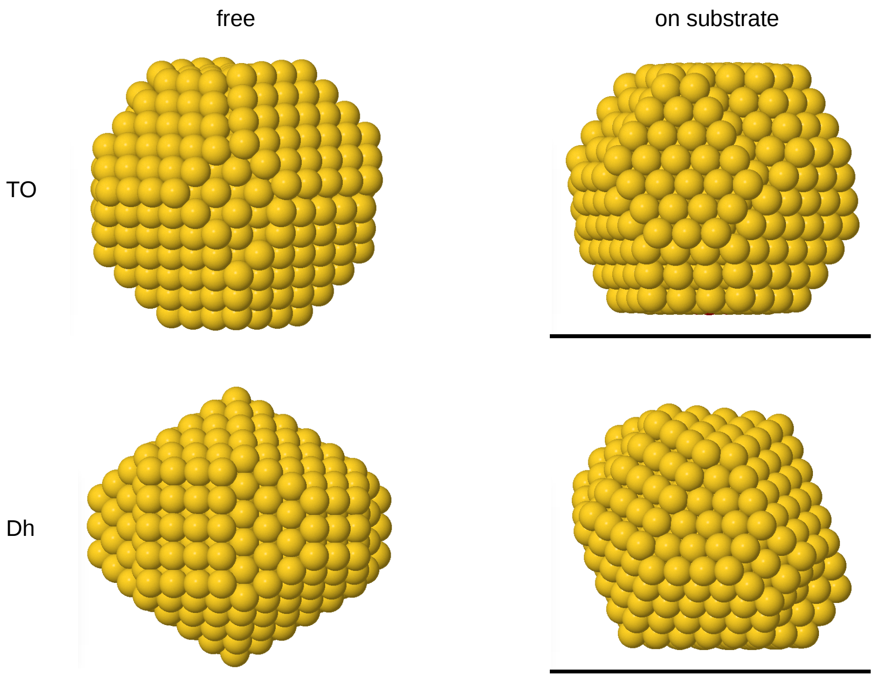

Supplementary Figure 4: Free and supported clusters. Supported clusters lean on their larger (111) facet.

Supplementary Table 1: Energy difference $\Delta E = E_{\text{fcc}} - E_{\text{Dh}}$ between the isomers for the different types of calculations.

	ΔE (eV)
gas phase	0.578
supported, P1	0.243
supported, P2	0.106

than the Dh, because the former presents larger (111) facets that can adhere to the surface. Thus it is a possible that the Dh structure might have slightly lower energy in the free cluster, but slightly higher energy on the surface.

Supplementary References

1. Calvo, F., Doye J. P. K. and Wales, D. J. Equilibrium properties of clusters in the harmonic superposition approximation. *Chem. Phys. Lett.* **366**, 176–183 (2002).

2. Baletto, F. and Ferrando, R. Structural properties of nanoclusters: Energetic, thermodynamic, and kinetic effects. *Rev. Mod. Phys.* **77**, 371–423 (2005).

3. Wells, D., Rossi, G., Ferrando, R. and Palmer R. E. Metastability of the atomic structures of size-selected gold nanoparticles. *Nanoscale* **7**, 6498–6503 (2015).

4. See for example *Condensed Matter Theories* Vol. 25, edited by Ludeña, E. V., Bishop R. F. and Iza, P. World Scientific, Singapore, 2011, pp. 45–46.

5. Luedtke, W. D. and Landman, U. Slip diffusion and Lévy flights of an adsorbed gold nanocluster. *Phys. Rev. Lett.* **82**, 3835–3838 (1999).

6. Lewis, L. J., Jensen, P., Combe, N. and Barrat, J.-L. Diffusion of gold nanoclusters on graphite. *Phys. Rev. B* **61**, 16084–16090 (2000).
